# Plantar Vein Thrombosis and Thrombophlebitis: An Uncommon Diagnosis in Emergency Medicine

**DOI:** 10.7759/cureus.91608

**Published:** 2025-09-04

**Authors:** Ian D Storch, Michael Carpenter, Kaleena Shirley

**Affiliations:** 1 Emergency Medicine, University of Florida College of Medicine – Jacksonville, Jacksonville, USA

**Keywords:** ankle joint swelling, medial ankle pain, medial foot pain, plantar vein thrombophlebitis, plantar vein thrombosis

## Abstract

Plantar vein thrombosis and plantar vein thrombophlebitis are not diagnoses typically made or considered in the emergency department. However, septic joints or gout are more commonly considered diagnoses. Early diagnosis of a septic joint is imperative, as there are many short-term and long-term consequences, such as sepsis, osteomyelitis, amputation, chronic arthritis, or joint replacement. Septic joint is a rare diagnosis and often only pursued with further diagnostic testing in the emergency department in patients with risk factors, such as our patient, who was on immunosuppressant medications. This case report describes the emergency department encounter of a patient with certain risk factors and physical exam findings that could be suggestive of a septic joint but was ultimately found to have a rare, yet less emergent, diagnosis.

## Introduction

Ankle pain is a common presenting complaint in the emergency department. While its incidence is often seen in traumatic settings with sprains and fractures, there are more than 600,000 visits annually for ankle pain [1]. In outpatient podiatry clinics, plantar heel and arch pain accounts for 11-15% of visits [2]. Meanwhile, plantar vein thrombosis and plantar thrombophlebitis are rare conditions, with an estimated 30 to 100 cases ever reported [2,3].

Plantar vein thrombosis and plantar thrombophlebitis are believed to be underdiagnosed, possibly due to misdiagnosis, as they share similar presentations with other more common diagnoses. Limited diagnostic capabilities could be another possible reason.

The most common presenting symptom is pain, especially while standing or walking. There is often associated swelling and sometimes erythema. Risk factors include, but are not limited to, localized trauma, recent surgery, hypercoagulable states, and even prolonged periods of sitting or standing [4]. Compressive ultrasonography (US) and MRI can make the diagnosis. However, the most common US study performed in emergency departments in the United States is a lower extremity US to rule out deep vein thrombosis (DVT) [3]. A lower extremity DVT US typically does not go past the proximal leg, so the plantar vein would not be visualized. Rarely would a compressive US of the vasculature in the foot and ankle be performed as part of a regular emergency department evaluation. While some emergency departments have an MRI available, many require transfer or admission to access an MRI.

## Case presentation

A 39-year-old male presents with left foot and ankle pain, worsening over the last three days. There was no trauma associated with this pain, but there is associated swelling. The patient’s past medical history is pertinent for autoimmune hepatitis requiring liver transplant, for which he takes tacrolimus and prednisone. The patient also has a history of ulcerative colitis for which he underwent a colectomy. The patient was already evaluated two days prior for the same symptoms, and the patient states the symptoms are getting worse. Initially, there was only a small area of tenderness just medial to the Achilles tendon. A DVT US and ankle X-ray were negative at that time. Since that visit, the patient has not been ambulating on the left lower extremity and has been following instructions for supportive care, including rest, ice, compression, and elevation. Despite this, the pain is now described as severe, and the ankle has a decreased range of motion. The pain is worse in the dependent position and better with elevation. The pain has also spread from the medial aspect to include the plantar aspect, and some erythema has developed. The patient denies any prior injuries or surgeries to that foot or ankle. Denies fevers, chills, nausea, vomiting, numbness, or weakness. Denies a history of gout. He has a history of pulmonary embolism (PE) after his colectomy, but no unprovoked DVT/PE. The patient also takes lisinopril for hypertension. The patient does not smoke.

His vital signs are within normal limits, except for a blood pressure of 172/116 mmHg. His physical exam is only remarkable for generalized swelling of all four extremities. Specifically for the left lower extremity, there is tenderness to palpation (TTP), erythema, and warmth to the medial aspect of the left ankle that extends onto the plantar aspect of the foot but not to the toes. He has strong pulses and normal capillary refill. His range of motion is limited, and he has pain with passive dorsi and plantar flexion and lateral deviation of the foot and ankle. No pain is experienced with passive stretching of the toes. The lateral ankle does not have TTP or erythema.

His emergency department evaluation included laboratory results, a bedside US, and ultimately an MRI. His laboratory results are reflected in Tables 1-4.

**Table 1:** Complete blood count * The result is outside the reference (normal) range. WBC: white blood cell count, RBC: red blood cell count, MCV: mean corpuscular volume, MCH: mean corpuscular hemoglobin, MCHC: mean corpuscular hemoglobin concentration, RDW: red cell distribution width, MPV: mean platelet volume, nRBC: nucleated red blood cell, NRBC: nucleated red blood cell

Test	Patient value	Reference range
WBC	12.46 (*)	4.5-11 THOU/CUMM
RBC	5.02	4.50-6.30 x10E6/UL
Hemoglobin	15.1	14.0-18.0 G/DL
Hematocrit	46.5	40.0-54.0%
MCV	92.6	82.0-101.0 FL
MCH	30.1	27.0-34.0 PG
MCHC	32.5	31.0-36.0 G/DL
RDW	14	12.0-16.1%
Platelet count	274	140-440 x10E3/UL
MPV	10.6	9.5-12.2 FL
nRBC	0	0.0-1.0%
Absolute NRBC count	0	10 x 9/L

**Table 2:** Complete blood count manual differential * The result is outside the reference (normal) range. Lymphs: lymphocytes, Eos: eosinophils, Basos: basophils, Monos: monocytes, Abs: absolute

Test	Patient value	Reference range
Neutrophil	78 (*)	34-73%
Bands	0	0-10%
Lymphs	5 (*)	25-45%
Monocytes	5	%
Eos	3	%
Basos	1	%
Metamyelocytes	0	≤0%
Myelocytes	0	≤0%
Promyelocytes	0	≤0%
Atypical lymphocytes	7.9	0-10%
Blasts	0	≤0%
Other cell percent	0	≤0%
Neutrophil Abs	9.72 (*)	1.4-7.5 X10E3/UL
Lymph Abs	1.64	0.90-3.00 THOU/CUMM
Monos Abs	0.66	0.20-0.90 X10E3/UL
Eos Abs	0.32	0.20-0.90 THOU/CUMM
Basos Abs	0.11 (*)	0.00-0.10 X10E3/UL
Immature	0	≤0.00 x10E3/UL
Granulocytes		-
Absolute		-

**Table 3:** Basic metabolic panel * The result is outside the reference (normal) range. CO2: carbon dioxide, BUN: blood urea nitrogen, CALC: calculated, EGFR: estimated glomerular filtration rate, CKD-EPI: chronic kidney disease epidemiology collaboration equation

Test	Patient value	Reference range
Sodium	137	135-145 MMOL/L
Potassium	4.2	3.4-4.5 MMOL/L
Chloride	103	98-107 MMOL/L
CO2	24	21-29 MMOL/L
Urea nitrogen	17	6-22 MG/DL
Creatinine	0.9	0.67-1.17 MG/DL
BUN/creatinine ratio	18.9	6.0-22.0 (CALC)
Glucose	130 (*)	71-99 MG/DL
Calcium	8.6	8.6-10.0 MG/DL
Osmolality calc	287.3	275.0-295.0
Anion gap	10	4-16 MMOL/L
EGFR (CKD-EPI)	111	≥60 ML/MIN/1.73M2

**Table 4:** Inflammatory markers * The result is outside the reference (normal) range. Sed rate: erythrocyte sedimentation rate, CRP: C-reactive protein

Test	Patient value	Reference range
Sed rate	23 (*)	0-15 MM/HR
CRP, high sensitivity	2.26	0.10-2.80 MG/L

**Figure 1:**
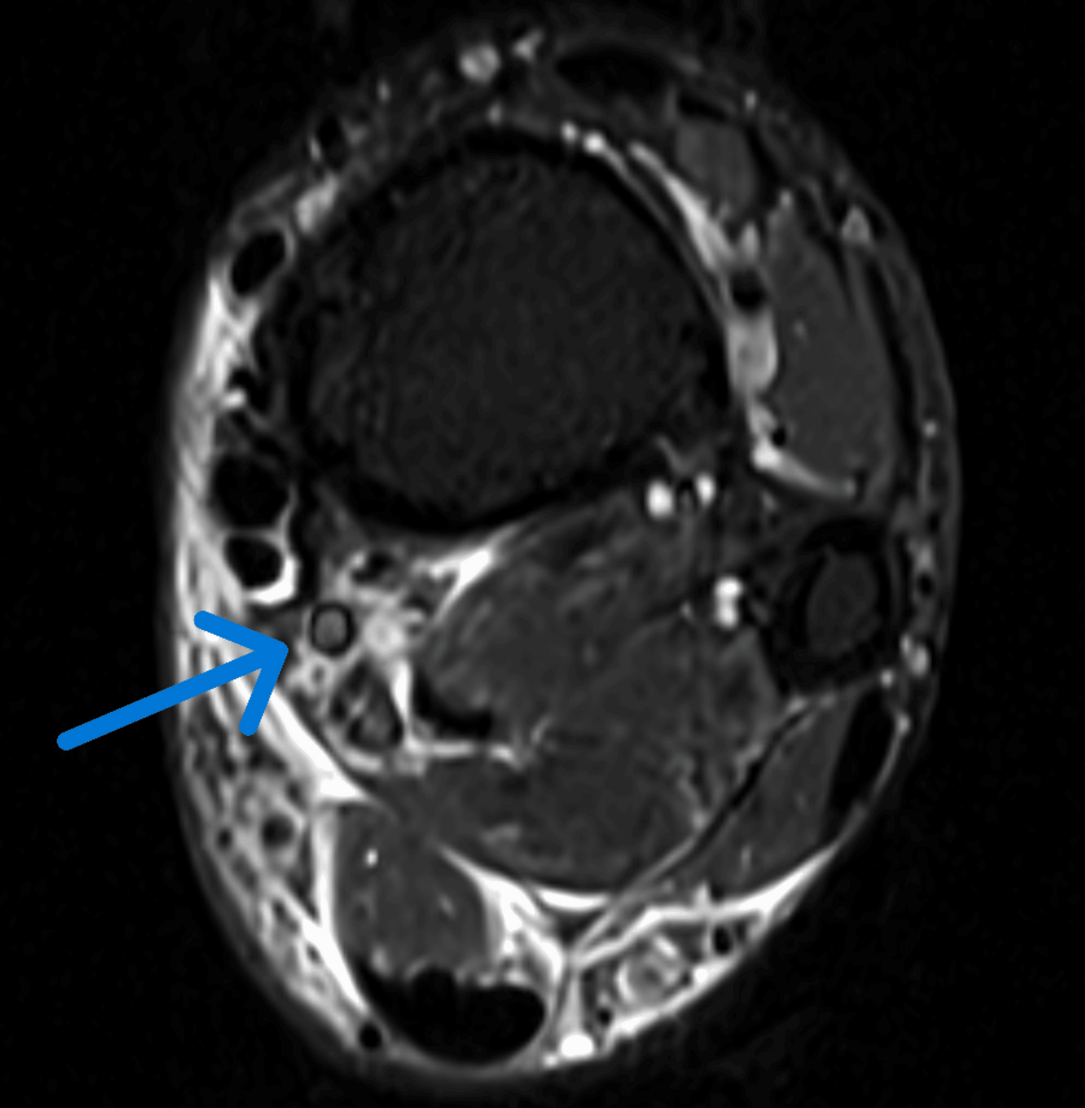
MRI revealing vein thrombosis in the distal leg Vein thrombosis in the distal leg, at the level of the distal tibia and fibula, just above the ankle.

**Figure 2:**
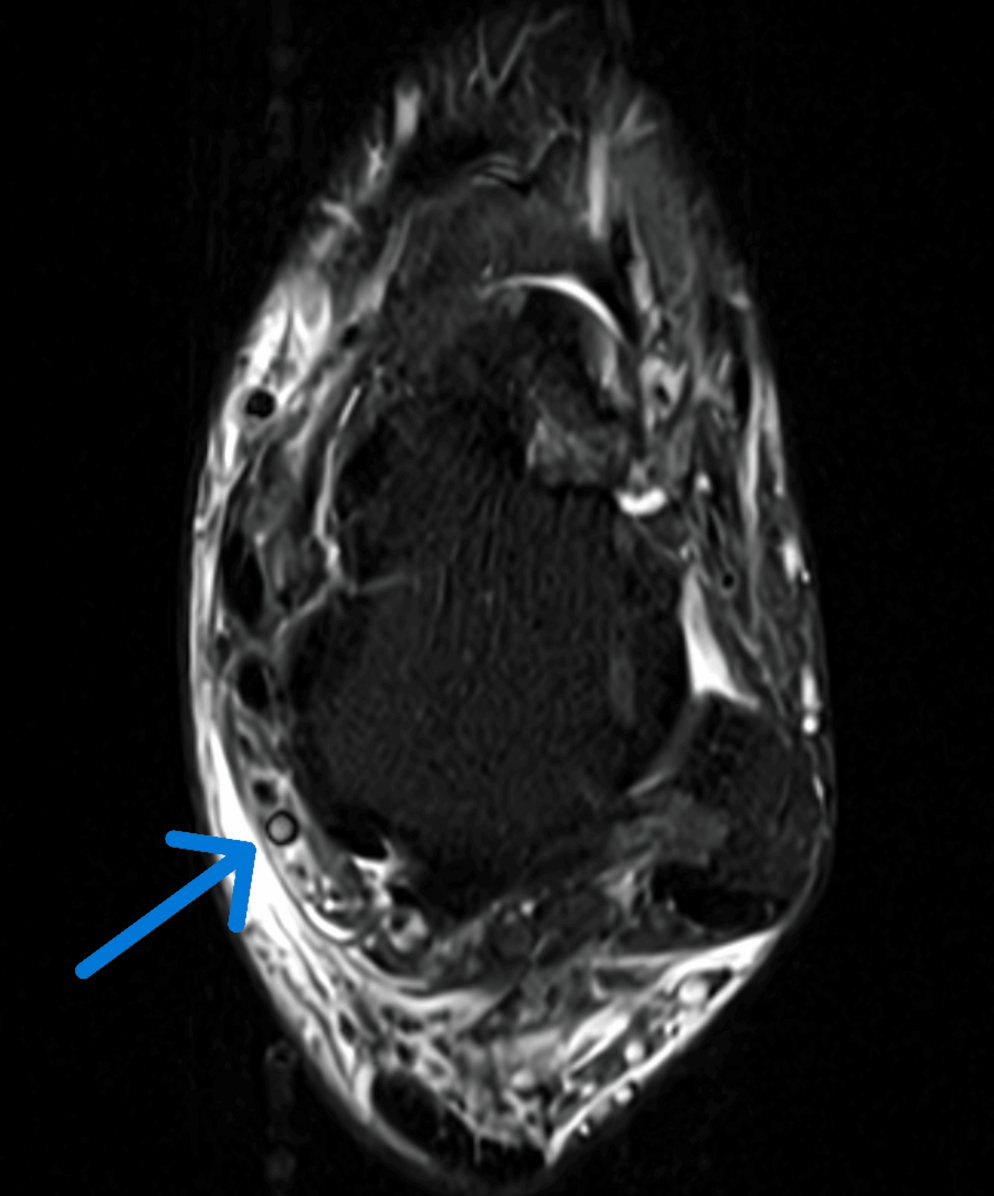
MRI revealing vein thrombosis near the ankle Vein thrombosis medial to the talus.

**Figure 3:**
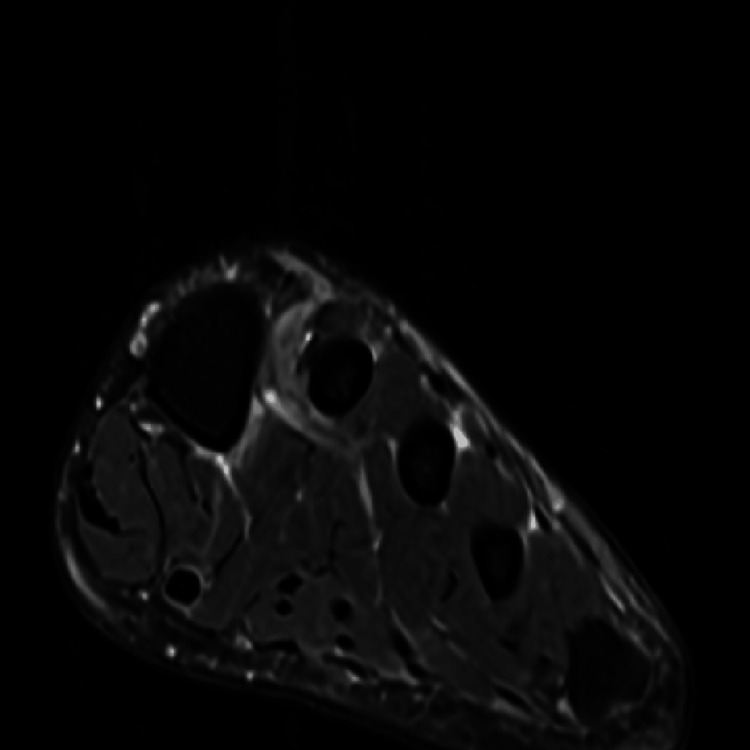
MRI revealing normal vasculature distal to the plantar vein Open venous structures without thrombus inferior to the metatarsals.

His bedside point-of-care musculoskeletal US at the site of maximal tenderness did not show a significant drainable effusion, only soft tissue cobblestoning. After considering the lab results in Tables 1-4 and the US results, a septic joint seemed less likely; however, a definitive diagnosis was still not established. This was an immunosuppressed patient with increasing unilateral ankle pain, erythema, swelling, warmth, and limited range of motion, presenting with worsening symptoms over three days. He was noted to have mild leukocytosis; however, he was also on chronic steroids (Table 1). An MRI was completed in the emergency department, and the images are shown in Figures 1-3.

Edema and mild regional post-contrast enhancement are seen within the medial flexor compartment, extending to the plantar aspects of the midfoot and hindfoot along the course of the plantar venous complex. These findings are suggestive of plantar thrombophlebitis, which could be confirmed with a vascular US examination if needed. No organized, drainable fluid collections or significant soft tissue enhancement are observed, suggesting an infectious process. No evidence of osteomyelitis is noted. Circumferential ankle edema is present, likely reactive to the above. Insertional tendinopathy of the posterior tibialis tendon is also observed.

## Discussion

Identifying the cause of atraumatic, nonspecific foot or ankle pain in the emergency department can be challenging. Overuse injuries, minor strains, or muscle inflammation are all common, but these conditions typically do not have satisfying binary radiological or other findings to confirm a medical cause or injury.

Plantar vein thrombosis is also challenging to diagnose. The two modalities best described by current literature are a focused US of the distal/plantar veins specifically or an MRI [5,6]. It is not evaluated in a typical lower extremity DVT US examination, which includes imaging of more proximal vasculature in the lower extremity, and MRIs are not routinely ordered for foot and ankle pain in the emergency department. The condition is rare enough that most clinicians are unlikely to have a high degree of suspicion that would prompt detailed imaging, such as an MRI or formal US, of the plantar veins.

In the case above, the patient returned for a second visit after unresolved, acute, unilateral pain. As noted, the patient did have a history of a single provoked PE following colectomy and autoimmune hepatitis, but no other clear hypercoagulable pathology.

Naturally, the primary concern when identifying any thrombus-related pathology is the risk of developing a DVT or, more importantly, a PE. A review of the limited number of case reports did not demonstrate any cases of progression to pulmonary embolus with conservative treatment, including non-steroidal anti-inflammatory drug therapy, non-weight-bearing periods, rest, and compression therapy [2]. Other cases have been treated with anticoagulant therapy; these unsurprisingly also did not progress to pulmonary embolus [7]. In several of these latter cases, the provocative insult was thought to be a physical strain of the foot, which may have been the case in this patient.

The number of cases of plantar vein thrombosis is relatively low, likely due in part to the difficulty in identification on imaging modalities, combined with a relatively low occurrence in general. It is unclear how many diagnoses are missed due to these limitations. It seems reasonable that the select patients in whom a plantar vein thrombosis is identified should be carefully evaluated for chest pain, shortness of breath, and other symptoms of deeper and more dangerous thromboembolic pathology. For isolated plantar vein thrombus or thrombophlebitis, the above conservative management appears to be adequate. However, anticoagulation is a reasonable option for prolonged symptoms or patients at higher risk of coagulopathy [2].

## Conclusions

Plantar vein thrombosis is a rare condition that can be challenging to diagnose in the emergency department. It typically presents with unilateral plantar foot and heel pain, redness, and swelling. Without a high index of suspicion, cases may remain undiagnosed unless targeted imaging such as US or MRI is performed. Awareness of this condition is therefore critical. In patients with a low likelihood of more emergent diagnoses, such as septic arthritis, conservative management for plantar vein thrombosis may be appropriate, with outpatient imaging recommended for confirmation. In this case, conservative treatment with aspirin, nonsteroidal anti-inflammatory drugs, and warm compresses was advised. The potential initiation of anticoagulation was discussed should symptoms persist or worsen.
